# The Clinical and Phenotypic Significance of Sub-Threshold JAK2 V617F Signals in Patients Classified as JAK2 V617F-Negative

**DOI:** 10.3390/cimb48101070

**Published:** 2026-10-16

**Authors:** Şerife Emre Ünsal, Hilal Akalın, Büşra Tan, Gülşah Polat Akyol, Aslıhan Kiraz

**Affiliations:** 1 Department of Hematology, Erciyes University, Kayseri 38280, Turkey; serifeemre1@gmail.com (Ş.E.Ü.); drgakyol@gmail.com (G.P.A.); 2 Department of Medical Genetics, Erciyes University, Kayseri 38280, Turkey; hakalin@erciyes.edu.tr (H.A.); busrademirors@erciyes.edu.tr (B.T.)

**Keywords:** JAK2 V617F mutation, allele burden, hematological neoplasms

## Abstract

Background: This study investigates the relationship between clinical and hematological parameters and sub-threshold positive findings in cases with suspected hematological malignancies subjected to Janus Kinase 2 (JAK2) V617F qPCR testing. Methods: Adult patients referred to the Medical Genetics Clinic from the Erciyes University Faculty of Medicine Hematology Department between 2022 and 2024 for JAK2 V617F analysis due to suspected hematological malignancy were evaluated in this retrospective, cross-sectional study. Registered data from 2394 patients were analyzed, and 173 cases with a JAK2 V617F allele burden below 0.1% but above the negative control value were included in the study. SPSS version 25.0 software was used for statistical analysis. Results: More than half of the patients (56.6%) had no definitive hematological diagnosis at initial testing, while 28.9% developed a hematological disorder during follow-up, including overt MPN in 13.3%. Significant differences across VAF groups were observed for leukocytosis (*p* = 0.004), neutrophilia (*p* = 0.030), and monocytosis (*p* = 0.024), with the highest frequencies in Group 3 (0.041–0.099%). ROC analyses identified closely clustered optimal cut-offs around 0.027–0.029%, whereas no independent predictor of higher sub-threshold allele burden emerged in multivariate analysis (*p* > 0.05). Conclusions: Even sub-threshold levels of the JAK2 V617F mutation may exhibit specific clinical manifestations and may be associated with constitutional symptoms and leukocytosis in particular. The study findings also showed a lower awareness of the JAK2 test in clinical practice, and that it is significantly employed in off-label cases. In order to accurately interpret low-allele-burden results, molecular findings must be evaluated in conjunction with hematological parameters and appropriate clinical indications. The findings underscore the importance of applying the JAK2 V617F PCR test selectively and based on clinical suspicion.

## 1. Introduction

In accordance with the 2022 World Health Organization (WHO) classification, myeloproliferative neoplasms (MPNs) are classified by evaluating their genetic, morphological, molecular, and clinical characteristics. This classification encompasses polycythemia vera, essential thrombocythemia, primary myelofibrosis, chronic myeloid leukemia (BCR-ABL1 positive), chronic neutrophilic leukemia, chronic eosinophilic leukemia, and mast cell disease. Although a diverse array of genetic mutations can be identified across these neoplasms, the Janus kinase 2 (JAK2) pV617F mutation is a prominent critical hallmark. The JAK2 V617F mutation, which enhances tyrosine kinase activity, is not present in all MPNs. It is detected in approximately 95% of polycythemia vera cases and 50–60% of essential thrombocythemia and primary myelofibrosis cases [1]. This mutation significantly impacts disease progression, the immunophenotype, and patient outcomes [2].

The advent of highly sensitive diagnostic techniques in clinical practice has enabled low-level clones to be detected down to variant allele frequencies (VAFs) of <1%. This has consequently necessitated a closer examination of low allele burdens, the exact clinical implications of which have yet to be fully defined [3].

In order to elucidate the detection and significance of low VAFs, previous studies have emphasized that the polymerase chain reaction (PCR) must precisely quantify the JAK2 V617F mutation, even at low levels, since this is critical for diagnosis, treatment monitoring, and prognosis in patients with MPNs [3].

According to the 2022 International Consensus Classification (ICC) guidelines, JAK2 V617F testing should achieve a sensitivity capable of detecting a mutant burden of at least ≈1%, any positivity below this level requiring rigorous analytical validation and clinical correlation [4]. This improved sensitivity offers a promising avenue for the early detection of minimal residual disease in patients with MPN and permits the accurate quantification of clonal hematopoiesis with low mutant allele burdens within the general population [5].

Low-level JAK2 positivities therefore require a comprehensive evaluation that integrates pre-analytical and analytical accuracy, the clinical context, and the appropriateness of the indication. Moreover, investigations exploring the association between allele burden and clinical phenotype, such as symptoms, thrombosis, and thrombocytosis, have yielded heterogeneous findings in the low-to-intermediate VAF spectrum [6].

The clinical significance of very low-level JAK2 V617F signals below the routine positivity threshold is still uncertain. In particular, it is unclear whether patients classified as JAK2 V617F-negative by conventional testing, but with a detectable signal close to the assay positivity threshold, may exhibit clinical or hematological features suggestive of an underlying myeloproliferative neoplasm (MPN). The present study therefore focused specifically on patients with JAK2 V617F allele burdens below the predefined positivity threshold of 0.1% and investigated whether the relative level of this sub-threshold signal was associated with MPN-related clinical manifestations, including splenomegaly and disease-related symptoms. In order to explore this relationship, the narrow sub-threshold VAF range was divided into three consecutive groups according to the magnitude of the detected JAK2 V617F signal.

## 2. Materials and Methods

### 2.1. Study Population

This retrospective, cross-sectional study evaluated the medical data for 2394 adult patients admitted to the Department of Hematology at the Erciyes University Faculty of Medicine, Türkiye, between 2022 and 2024. The study timeframe (2022–2024) was specifically selected because a standardized, uniform qPCR kit lot and consistent negative control baseline (ipsogen^®^ JAK2 MutaQuant^®^) were maintained throughout this period. Subsequent changes in commercial assay platforms and reference controls after 2024 would have introduced analytical heterogeneity; data collection was deliberately restricted to this uniform pre-analytical period to ensure optimal analytical accuracy. In addition, medical records were reviewed longitudinally during the follow-up period to evaluate whether patients developed an overt MPN over time. The study cohort consisted of individuals with clinical suspicion of hematological malignancy who were subsequently referred to the Department of Medical Genetics for JAK2 V617F mutation testing. The primary objective was to perform a comprehensive analysis of the subset of patients presenting with sub-threshold positivity.

In all evaluated cases, molecular and laboratory testing was performed in the setting of persistent leukocytosis and/or erythrocytosis, rather than transient abnormalities. Patients with active acute infections or manifest secondary inflammatory conditions were excluded from evaluation. Furthermore, potential non-neoplastic and reactive etiologies contributing to leukocytosis and erythrocytosis, specifically active cigarette smoking, chronic obstructive pulmonary disease (COPD), and obstructive sleep apnea syndrome (OSAS), were systematically reviewed and documented from the electronic medical records.

### 2.2. Molecular Evaluation

The JAK2 V617F mutation analysis was conducted in strict compliance with the manufacturer’s instructions accompanying the ipsogen^®^ JAK2 MutaQuant^®^ kit (Qiagen, Hilden, Germany). In accordance with the protocol guidelines, patients exhibiting a JAK2 V617F allele burden of ≥0.1% were classified as positive and subsequently excluded from the study. Conversely, samples demonstrating an allele burden below the 0.1% threshold, yet remaining above the negative control value, were included for detailed evaluation.

Quantitative assessment of the somatic JAK2 V617F (c.1849G>T) mutation in genomic DNA was performed using the ipsogen JAK2 MutaQuant real-time PCR assay according to the manufacturer’s protocol. The assay provides a limit of detection of 0.061%, achievable with sample inputs of at least 10,000 total JAK2 copies (wild-type and mutant alleles combined). Quantification was standardized against a four-point plasmid dilution series for both mutant and wild-type alleles, with the mutant allele burden expressed as the percentage of JAK2 V617F relative to total JAK2. The positive control should yield a JAK2 V617F percentage of greater than 99.9%. The negative control should yield a JAK2 V617F percentage of less than 0.1%.

In every experimental run, each patient sample was assayed in parallel using specific primers and probes for both the wild-type and mutant alleles. Rigorous quality control was maintained by including the kit-provided positive and negative control samples in each analytical run, and patient results were evaluated and normalized relative to these controls. Furthermore, a no-template control containing only the master mix without a DNA template was incorporated into every run to monitor and exclude potential reagent or environmental contamination.

Patients with detectable JAK2 V617F signals ranging from 0.01% to <0.1%, which were below the predefined positivity threshold of the assay, were included in the sub-threshold analysis. Since the primary objective was to determine whether the magnitude of the JAK2 V617F signal within this narrow sub-threshold range was associated with clinical manifestations, patients were stratified into three consecutive groups according to their measured VAFs. These groups represent relative levels within the sub-threshold range and should not be interpreted as conventional low-, intermediate-, or high-VAF categories used for MPN classification. The group boundaries were predefined according to the distribution of VAF values in the study population.

### 2.3. Statistical Methods

Statistical analyses were performed using SPSS version 25.0 software. Correlations between the JAK2 V617F allele burden and hematological parameters were assessed using Spearman’s rank correlation coefficient. Bonferroni correction was applied in order to mitigate the risk of Type I errors during multiple comparisons. A multivariate logistic regression model was employed to determine the independent predictors of a high allele burden. The diagnostic capability of the allele burden to predict clinical and hematological manifestations was evaluated through receiver operating characteristic (ROC) curve analysis, using the Youden index to identify optimal cut-off thresholds. Statistical significance was strictly defined as *p* < 0.05.

### 2.4. Ethics Committee Approval

Approval for this study was obtained from the Ethics Committee of Erciyes University Faculty of Medicine (Approval No: 2026/87, Date: 18 February 2026). The study was conducted in accordance with the principles of the Declaration of Helsinki.

## 3. Results

**Figure 1:**
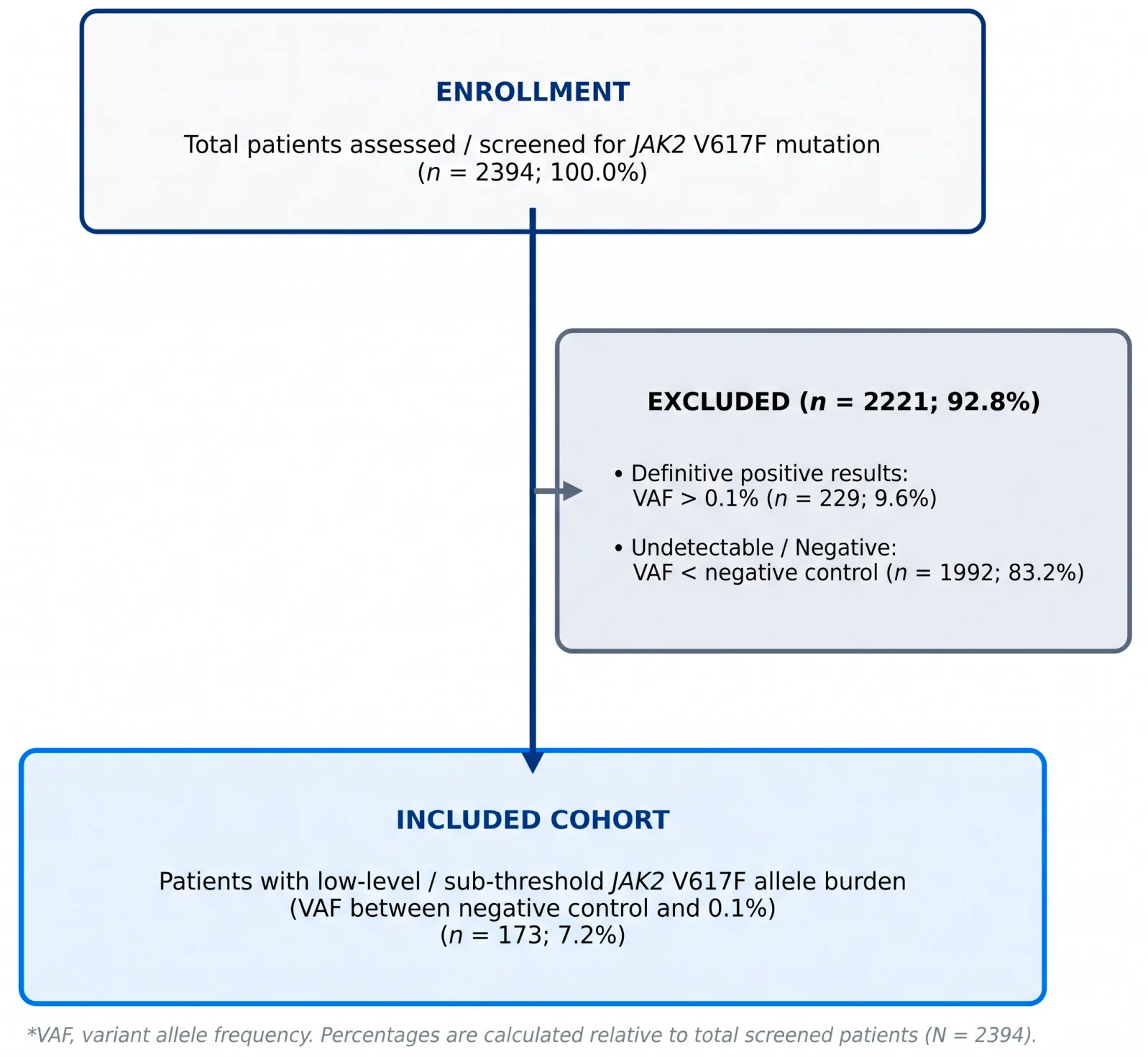
Study flow diagram of patient selection and cohort stratification. A total of 2394 consecutive patients screened for the JAK2 V617F mutation via quantitative real-time PCR were evaluated. Patients with overt positivity exceeding the established analytical threshold (VAF > 0.1%, *n* = 229) and those with allele burdens below the negative control baseline (*n* = 1992) were excluded. The final analysis included 173 patients harboring low-level/sub-threshold JAK2 V617F allele burdens (VAF between the negative control limit and 0.1%).

A total of 2394 patients undergoing JAK2 V617F screening were evaluated. Of these, 2221 patients were excluded: 229 had definitive positive results exceeding the conventional 0.1% diagnostic threshold (VAF > 0.1%), and 1992 yielded values below the negative control baseline. Consequently, 173 patients presenting with sub-threshold/low-level JAK2 V617F allele burdens (ranging between the assay’s negative control threshold and 0.1%) were enrolled in the final study cohort (Figure 1).

The study cohort consisted of 173 patients, with a mean age of 51 ± 17.1 years, 73.4% men and 26.6% women. No comorbidities were present in 141 (81.5%). Pulmonary disease was present in 16 (9.2%) of the patients with comorbid conditions, 9 (5.2%) had solid organ malignancies in nine (5.2%), and OSAS in three (1.7%). Congenital hemophilia, thalassemia major, immune thrombocytopenia (ITP), and chronic liver disease were also present in one patient (0.6%) each. When comorbidities and hematological disorders developing during follow-up were evaluated, 98 patients (56.6%) had no additional comorbid conditions. Obstructive pulmonary diseases, COPD and asthma, were present in 16 patients (9.2%). During the follow-up period, 50 patients (28.9%) were observed to have developed a hematological disorder or malignancy. However, only 23 patients (13.3%) were diagnosed with a JAK2 V617F-associated MPN. Seventeen of these patients were diagnosed with polycythemia vera, four with myelofibrosis, and two with essential thrombocythemia. Other hematological malignancies diagnosed during follow-up included chronic myeloid leukemia (CML), myelodysplastic syndrome (MDS), lymphoma, and acute leukemia.

A value of 0.029% obtained from the ROC analysis was adopted as the cut-off for the JAK2 V617F allele burden. The patients with subthreshold JAK2 V617F signals were divided into three groups according to the magnitude of the detected allele burden within the 0.01–0.1% range, ensuring a similar patient distribution across the groups, VAF Group 1 (0.010–0.020%), VAF Group 2 (0.021–0.040%), and VAF Group 3 (0.041–0.099%) allele burdens. Based on this classification, 24.2% (*n* = 42) of the patients were in VAF Group 3, 40.5% (*n* = 70) in VAF Group 2, and 35.3% (*n* = 61) in VAF Group 1. The mean leukocyte count in the entire cohort was 12.8 × 10^3^/µL (min:1.38; max:335), the mean hemoglobin level was 15.26 g/dL (min:5.5; max:24.1), and the mean platelet count was 272 × 10^3^/µL (min:2.5; max:1828).

Following the exclusion of patients diagnosed with hematological neoplasms other than MPNs (with the exception of CML), the distribution of patients among the allele burden groups was 42.3% in Group 1, 26.9% in Group 2, and 30.8% in Group 3 (*p* = 0.285) (Table 1).

**Figure 2:**
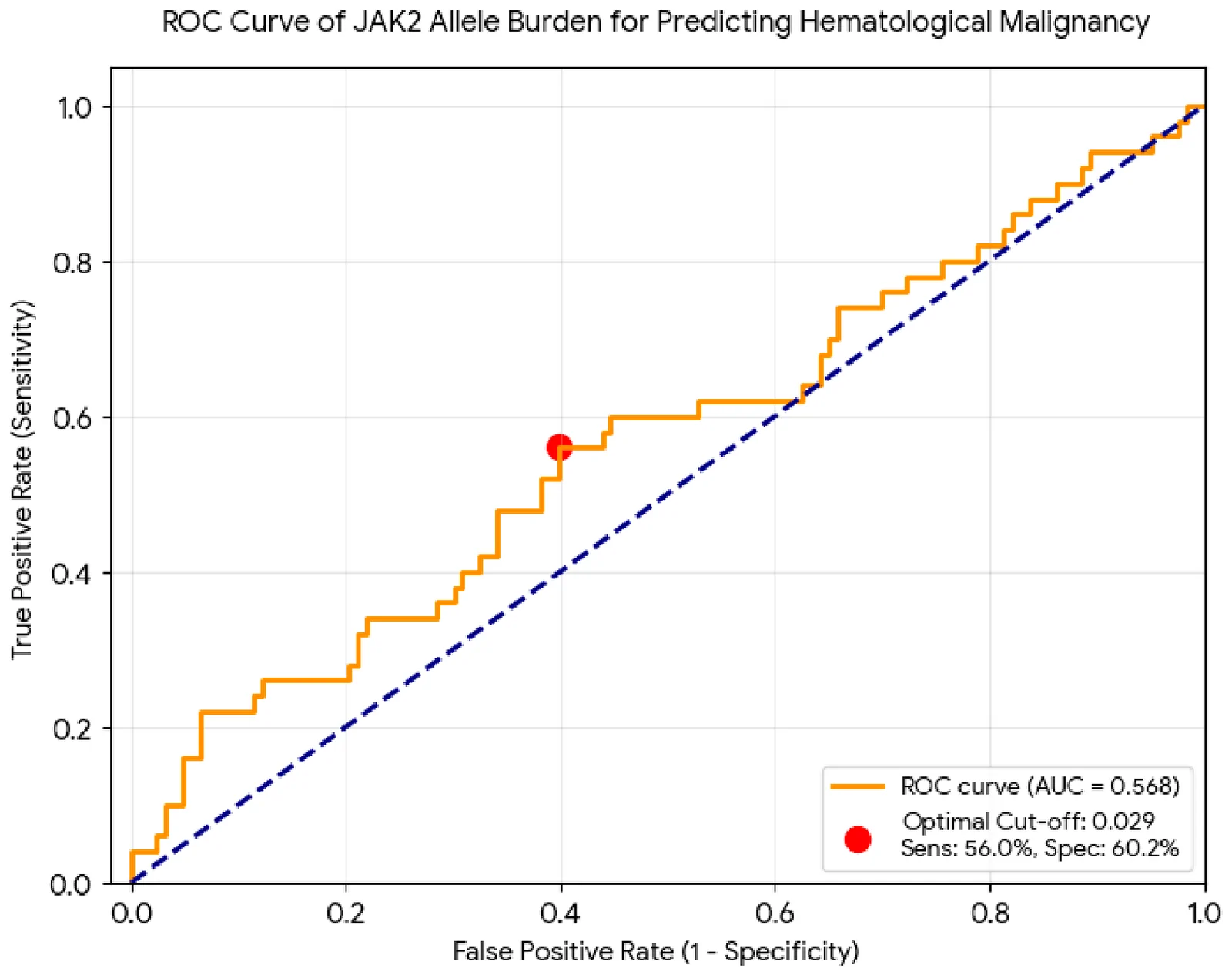
ROC curve analysis of the JAK2 V617F allele burden for predicting hematological malignancies. The optimal cut-off value was calculated as 0.029% using the Youden index (AUC = 0.568, Sensitivity: 56.0%, Specificity: 60.2%).

ROC curve analysis was performed to evaluate the diagnostic performance of the JAK2 V617F allele burden in predicting the presence of a hematological malignancy. Based on the Youden index, the optimal cut-off value was calculated as 0.028%. This statistically derived ideal threshold aligned closely with the median allele burden (0.026%) in our patient cohort, which was adopted as the methodological reference in this study. Furthermore, the weak discriminatory power indicated by the AUC value (0.568) confirms that hematological malignancies can also be observed in patients with low allele burdens (as demonstrated, for instance, in 42% of cases with erythrocytosis) (Figure 2).

Notably, 56.4% of the patients exhibiting constitutional symptoms had an allele burden above the established cut-off threshold. In addition, the prevalence of constitutional symptoms was markedly higher in Group 3 (VAF Group 1: 47.5%, VAF Group 2: 61.4%, VAF Group 3: 71.4%), the overall difference being statistically significant (*p* = 0.042). However, none of the post hoc pairwise comparisons remained statistically significant following Bonferroni correction (adjusted significance threshold, *p* < 0.0167). The pairwise *p*-values were 0.469 for Group 1 versus Group 2, 0.036 for Group 1 versus Group 3, and 0.131 for Group 2 versus Group 3. In addition, although splenomegaly was more common in Group 3, no statistically significant difference was observed in the frequency of the condition across the three VAF groups (26.4% in Group 1, 16.4% in Group 2, and 30.6% in Group 3; *p* = 0.807) (Table 1).

**Figure 3:**
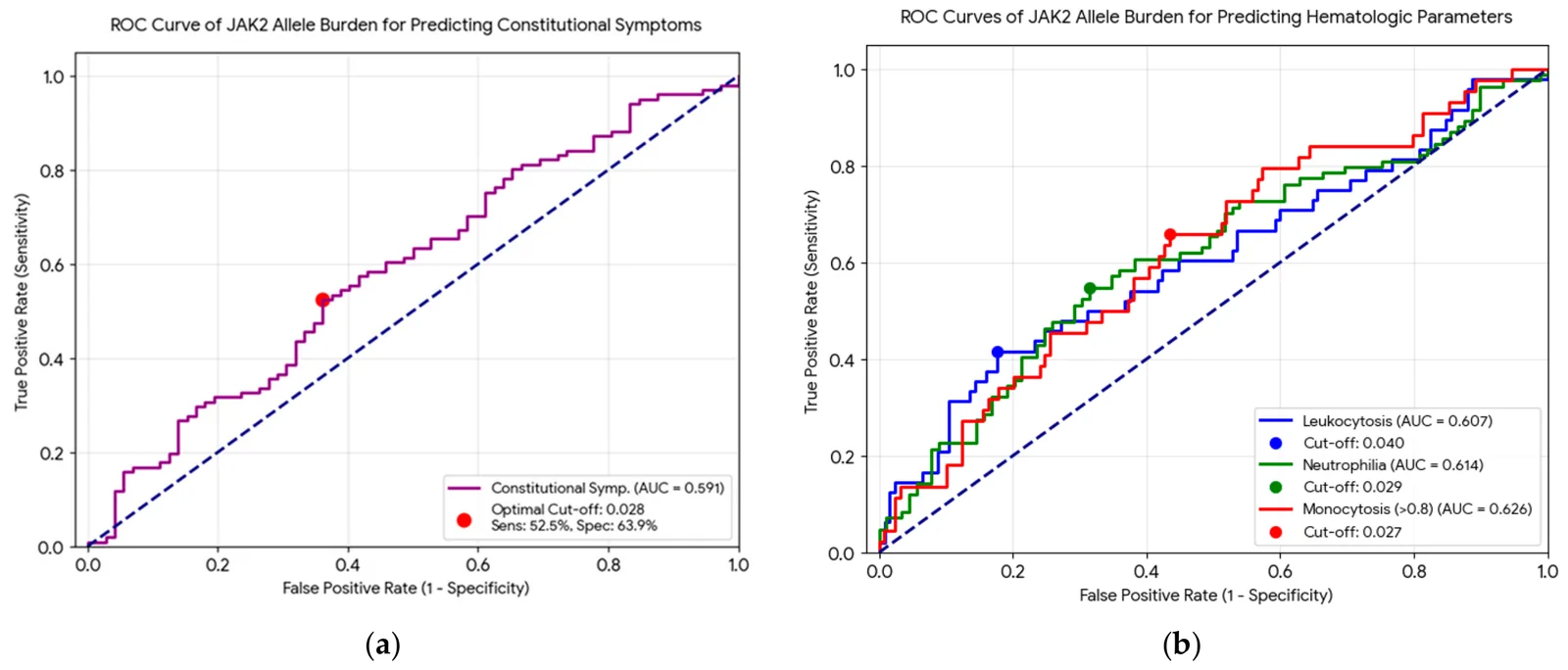
(**a**) ROC curve analysis of the JAK2 V617F allele burden for predicting constitutional symptoms (AUC = 0.591, Cut-off: 0.028%). (**b**) Multiple ROC curve analyses of the JAK2 V617F allele burden for predicting hematological parameters (leukocytosis [cut-off: 0.040%], neutrophilia [cut-off: 0.029%], and monocytosis [cut-off: 0.027%]).

Although ROC curve analyses identified mathematically optimal threshold values for constitutional symptoms and hematological parameters, clustered within the range of 0.026–0.029%, the corresponding AUC values remained modest (0.568–0.626). Sensitivity and specificity values were also predominantly within the range of 50–64%. Although this finding may suggest that a specific mutation burden level might serve as a threshold for the emergence of symptoms, from a classical biostatistical perspective, these AUC values fall within the category of poor discrimination (Figure 3).

Baseline continuous hematological counts, including white blood cell count, hemoglobin, platelet count, and leukocyte differentials, were comparable across the three sub-threshold VAF groups, showing no statistically significant differences (all *p* > 0.05; Table 2).

Evaluation of the hematological parameters revealed leukocytosis in 27.7% of the patients, erythrocytosis in 61.8%, thrombocytosis in 12.7%, lymphocytosis in 26.0%, neutrophilia in 48.6%, monocytosis in 26.0%, and eosinophilia in 26.0%. Overall comparisons across the three JAK2 V617F VAF groups revealed statistically significant differences in the incidences of leukocytosis (Pearson’s chi-square test, *p* = 0.004), neutrophilia (*p* = 0.030), and monocytosis (*p* = 0.024). Post hoc pairwise comparisons were subsequently performed with Bonferroni correction for three comparisons per variable (adjusted significance threshold, *p* < 0.0167). For leukocytosis, significant differences were observed between Group 1 and Group 3 (*p* = 0.009) and between Group 2 and Group 3 (*p* = 0.002), while no significant difference was observed between Group 1 and Group 2 (*p* = 0.681). For neutrophilia, only the comparison between Group 1 and Group 3 remained significant after Bonferroni correction (*p* = 0.008), whereas the comparisons between Group 1 and Group 2 (*p* = 0.211) and between Group 2 and Group 3 (*p* = 0.106) were not significant. Similarly, for monocytosis, a significant difference was observed only between Group 1 and Group 3 (*p* = 0.007), while no significant differences were observed be-tween Group 1 and Group 2 (*p* = 0.057) or Group 2 and Group 3 (*p* = 0.296) (Table 3).

Forty-two percent of the patients with a JAK2 V617F allele burden below the cut-off value, yet presenting with erythrocytosis, were diagnosed with a hematological malignancy (*p* = 0.001). This suggests that erythrocytosis represents a powerful clinical marker for hematological malignancy even at low allele burden levels, although this relationship may not be directly explained by the allele burden quantity alone.

Prior to the multivariate analysis, the relationships between the hematological parameters were evaluated using Spearman correlation analysis. As anticipated, statistically highly significant positive correlations were detected between the presence of leukocytosis and both neutrophilia (r = 0.535, *p* < 0.001) and monocytosis (r = 0.616, *p* < 0.001). Similarly, a significant positive correlation was observed between neutrophilia and monocytosis (r = 0.389, *p* < 0.001).

Variables that exhibited a significant association with VAF Group 3 in univariate analyses, namely leukocytosis, neutrophilia, monocytosis, and constitutional symptoms, were reevaluated by inclusion in a multivariate logistic regression model together with age and sex. Although the overall analysis was significant (*p* = 0.010), none of the parameters included in the model emerged as statistically significant independent predictors of a VAF Group 3. While leukocytosis (odds ratio [OR]: 2.414, 95% confidence interval [CI]: 0.820–7.106, *p* = 0.110) and the presence of constitutional symptoms (OR: 2.122, 95% CI: 0.957–4.706, *p* = 0.064) tended to increase the risk of a VAF Group 3, these did not reach statistical significance. This suggests that the significances observed in univariate analyses stemmed from the positive correlations among these hematological parameters (Figure 3a,b).

ROC analysis applied for hematological parameters revealed that the predictive power of the allele burden for monocytosis (AUC = 0.626) and neutrophilia (AUC = 0.614) was relatively higher than that for leukocytosis (AUC = 0.607). While the optimal cut-off values for monocytosis and neutrophilia were 0.027% and 0.029%, respectively, a higher mutation threshold (0.040%) was required for the emergence of leukocytosis (Figure 3b).

In the comparative analysis of patients with and without clinical indications for JAK2 V617F PCR testing, JAK2 test requests were evaluated as indication-compliant in 31 of the 35 patients (88.6%) with splenomegaly. Notably, requests for JAK2 analysis were significantly more prevalent in the presence of splenomegaly (Pearson χ^2^ *p* = 0.004; Fisher’s exact *p* = 0.003). Furthermore, in terms of the correlation between constitutional symptoms and indication-compliant testing, the analysis was conducted significantly more frequently among symptomatic patients, with a 77.2% (78/101) utilization rate (Pearson χ^2^ *p* = 0.008; Fisher’s exact *p* = 0.012).

Analysis of the association between hematological diagnosis and indication-compliant JAK2 testing revealed that 88% (44/50) of the patients were diagnosed with a hematological disorder as a result of these requests, with a significantly higher rate of indication-adherent testing (Pearson χ^2^ *p* = 0.001; Fisher’s exact *p* = 0.001).

The JAK2 V617F PCR testing rate among patients diagnosed with obstructive lung disease was 18.3%, the analysis being performed significantly more frequently as an off-indication request (Pearson χ^2^ *p* = 0.004; Fisher’s exact *p* = 0.008). Similarly, a robust correlation was identified between smoking status and the indication for JAK2 testing; the test was significantly more frequently requested without a proper clinical indication in 37.5% (6/16) of the smoking patients (Pearson χ^2^ *p* < 0.001; Fisher’s exact *p* < 0.001).

## 4. Discussion

This study explored the association between very low-level, sub-threshold JAK2 V617F allele burdens (defined as <0.1%) and distinct clinical and hematological profiles, as well as the utilization patterns and diagnostic yield of PCR testing in clinical practice. An important aspect of this research is that the observed allele burdens were substantially below the levels typically discussed in the context of established MPNs. The three VAF groups analyzed in this study should not therefore be interpreted as conventional low-, intermediate-, or high-VAF categories. Rather, the analysis was designed to examine whether the relative magnitude of a JAK2 V617F signal remaining below the routine positivity threshold is of clinical relevance.

The study findings suggest that even among patients classified as JAK2 V617F-negative by standard threshold-based testing, those with signals closer to the positivity threshold may exhibit clinical features compatible with an MPN phenotype. This raises the possibility that the quantitative level of a sub-threshold signal can provide clinically relevant information not captured by a simple positive/negative classification. However, the results also indicate that although a very low JAK2 V617F allele burden correlates with specific hematological entities, its reliability as a solitary diagnostic determinant is limited.

According to 2016 WHO criteria, JAK2 V617F molecular assays are required to be mutation-specific and to exhibit adequate sensitivity to identify clinically relevant low-level allele burdens essential for a diagnosis of MPN [5]. Similarly, the ICC guidelines recommend a meticulous interpretation of JAK2 V617F variant allele frequencies falling below 0.1% [4]. Our findings support the clinical relevance of allele burdens below this threshold in patients with suspected MPN, since these low-level signals were associated with distinct clinical and hematological features. In particular, the consistent associations between allele burden and several hematological parameters and symptomatic manifestations suggest that the magnitude of the JAK2 V617F signal may reflect clinically relevant phenotypic differences even within the sub-threshold range. Spearman correlation analysis further demonstrated moderate positive correlations between leukocytosis and both neutrophilia and monocytosis (r = 0.535 and r = 0.616, respectively; *p* < 0.001 for both). These findings are consistent with the observed association between sub-threshold JAK2 V617F signals and myeloid hematological features. However, these correlations should not be interpreted as evidence of a causal relationship. Rather, they suggest that low-level JAK2 V617F signals and alterations in myeloid lineage parameters may coexist within the same clinical phenotype.

A recent study demonstrated that low and very low JAK2 V617F mutant allele burdens (VAFs) may differ in terms of clinical course and clonal behavior. Among the patients who did not initially meet the diagnostic criteria for MPN, 72% of those in the very-low VAF group (0.06–<0.1%) and 50% of those in the low-VAF group (0.1–1%) remained below the diagnostic threshold, while the development of overt MPN remained limited in both groups during a median follow-up period of approximately 21 months. During clonal follow-up, complete disappearance of the mutation was observed in 33% of patients in the very-low VAF group, compared with only 7% of those in the low-VAF group. These findings suggest that clonal persistence and expansion at low VAF levels may vary considerably among patients and support the potential value of long-term monitoring [7].

This study not only evaluated the clinical and hematological features associated with sub-threshold JAK2 V617F signals, but also provided follow-up data for these patients. The primary aim was to determine whether low-level signals below the routine positivity threshold were associated with clinical and hematological findings. During the follow-up period, however, overt MPN developed or MPN was diagnosed in 23 of the 173 patients (13.3%). This finding is consistent with the biological heterogeneity and variable clonal persistence reported by Alyamany et al. among patients with very low VAF values ranging from 0.06% to <0.1% [7]. Nevertheless, differences in follow-up duration and study design should be considered when comparing these findings. Our results suggest that sub-threshold JAK2 V617F signals should not simply be regarded as technical background noise but may, in some patients, reflect an underlying clonal process. Therefore, rather than interpreting the JAK2 V617F signal in isolation, clinical and hematological findings should be considered together, and appropriate hematological follow-up should be maintained in these patients.

Clonal hematopoiesis of indeterminate potential (CHIP) is generally defined by the presence of somatic mutations with a VAF of ≥2% in the absence of diagnostic criteria for a hematological malignancy. Since the prevalence of clonal hematopoiesis increases substantially with age, the interpretation of very low-level JAK2 V617F signals should take into account the age distribution and clinical characteristics of the study population. In the present cohort, the mean age was 51 ± 17.1 years, and JAK2 V617F VAF levels were substantially below the conventional CHIP threshold. Nevertheless, constitutional symptoms and hematological findings suggestive of myeloid activity were observed in some patients despite VAF levels below 0.1%. These observations raise the possibility that, in some patients, very low-level JAK2 V617F signals may reflect an underlying clonal process or subclinical myeloproliferative activity, rather than a clinically silent state of clonal hematopoiesis. However, previous findings indicate that clones with very low VAF levels may disappear over time, whereas clones with VAFs between 0.1% and 1% may be more likely to persist [7]. These observations emphasize the need for further investigation of the clinical significance of JAK2-mutant clones with VAFs below 2%, rather than suggesting that the conventional CHIP threshold should be revised on the basis of the available evidence. Sub-threshold JAK2 V617F positivity should not therefore be automatically classified as CHIP; instead, it should be interpreted in conjunction with clinical findings, bone marrow histopathology, and longitudinal follow-up [8,9,10].

One of the most striking findings of this study is that the optimal allele burden thresholds for predicting hematological abnormalities and constitutional symptoms were clustered within a very narrow range (0.026–0.029%) according to the ROC analysis. This indicates that as the allele burden increases, the probability of systemic symptoms also rises. Meta-analyses in the literature support this finding by showing that patients with a higher JAK2 V617F allele burden have a significantly greater likelihood of developing pruritus. Research has reported that a high allele burden is strongly linked to symptoms and clinical features in patients with MPN [6]. Although the JAK2 V617F variant fraction in the current study was below 0.1%, the findings showed that constitutional symptoms can manifest even at these low allele burden levels.

Multivariate logistic regression analysis revealed that the variables of leukocytosis, neutrophilia, and monocytosis, identified as significant at univariate analyses, overshadowed one another in predicting a high allele burden (*p* > 0.05). This statistical finding confirms that changes in hematological parameters associated with a very low allele burden do not arise from an isolated lineage, but rather reflect a holistic and simultaneous clonal stimulation within the bone marrow. Methodologically, however, the relatively wide 95% CIs observed in our models (e.g., OR: 2.414, 95% CI: 0.820–7.106 for leukocytosis) strongly suggest that limited statistical power, resulting from cohort stratification across three narrow VAF subgroups, also contributed to the loss of statistical significance. Both biological colinearity and sample size constraints should therefore be acknowledged as plausible contributors to these findings.

Although the rate of thrombocytosis was higher in the group with a greater JAK2 V617F allele burden (0.041–0.099%), this difference was not statistically significant. Previous studies have reported a positive association between a higher JAK2 V617F allele burden and thrombosis; however, this association cannot be explained solely by platelet counts, and these counts may even be lower in patients with higher allele burdens [6]. This finding has been attributed to a shift from thrombopoiesis toward myelopoiesis. Nevertheless, the studies cited above primarily included patients with classical polycythemia vera and relatively high allele burdens and are therefore not directly comparable with the very low allele burden range (<0.1%) investigated in the present research. Accordingly, the absence of a significant association between platelet count and JAK2 V617F allele burden in our low-VAF cohort should be interpreted in the context of these differences in patient populations and disease biology.

Similarly, a previously cited study demonstrated a strong positive association between JAK2 V617F allele burden and splenomegaly [6]. In contrast, no significant association between allele burden and splenomegaly was observed in the present cohort. This discrepancy may partly reflect methodological factors, including the limited sample size and the absence of standardized spleen-size assessment in all patients. The relationship between very low JAK2 V617F allele burdens and splenomegaly therefore remains uncertain and warrants evaluation in larger cohorts with systematic assessment of spleen size.

JAK2 V617F PCR analysis results must be evaluated within the framework of clinical, morphological, hematological, and other laboratory findings. This approach requires that mutation analysis be requested only in case of suspicion compatible with clinical findings and that the results be interpreted in light of the patient’s overall clinical status [11]. The present study thus highlights a significant practical issue in the clinical use of molecular diagnostic tests. The JAK2 test was requested in the absence of a specific diagnosis for approximately half the cases (56.6%) included in this study, and was frequently requested unnecessarily in cases of secondary polycythemia, such as obstructive lung diseases (COPD/asthma). Conversely, the findings also demonstrated that in cases of suspected hematological disease, requests were made with appropriate indications.

High-sensitivity and high-specificity JAK2 V617F analysis methods not only permit the early detection of minimal residual disease, but may also contribute to the earlier identification of patients with low allele burdens [3]. Indeed, the JAK2 V617F mutations with low allele burdens included in the present study appear to be clinically significant, suggesting that JAK2 positivity rates among cases with a hematological diagnosis may be higher than previously reported in the literature.

### Limitations

This study has a number of limitations. The retrospective, single-center design and limited longitudinal follow-up preclude definitive conclusions regarding the natural history and potential clonal expansion of sub-threshold JAK2 V617F positivity. In addition, the absence of bone marrow biopsy in all cases limited the correlation of molecular findings with histopathological features, while the lack of CALR and MPL testing restricted comprehensive molecular characterization.

The presence of non-malignant pulmonary diseases, including COPD, asthma, and OSAS, as well as a history of smoking, represents another important potential confounding factor in our cohort. These conditions may contribute to secondary erythrocytosis, reactive leukocytosis, and systemic symptoms through chronic hypoxia and low-grade inflammation. Some of the observed hematological and symptomatic findings, particularly among patients who underwent JAK2 V617F testing in the absence of a specific hematological diagnosis, may therefore have been related, at least in part, to these reactive processes rather than to the sub-threshold clone itself. Prospective studies with detailed characterization of cardiopulmonary comorbidities are now needed to better delineate the biological and clinical significance of these low-level signals.

Finally, the relatively modest sample size within the stratified sub-threshold VAF groups was reflected in the wide confidence intervals observed in the multivariable logistic regression models. This limitation reduced the statistical precision of the estimated associations, particularly for leukocytosis and constitutional symptoms. Larger, preferably multicenter studies are now needed to validate these associations and provide more precise estimates of the clinical significance of sub-threshold JAK2 V617F signals.

## 5. Conclusions

In conclusion, the findings of this study suggest that JAK2 V617F signals below the 0.1% threshold (<0.100%), particularly when interpreted in conjunction with hematological data, may be associated with significant hematological abnormalities and constitutional symptoms, and that 13.3% of patients developed overt MPN during follow-up. However, the presence of a sub-threshold JAK2 V617F signal alone does not establish an MPN diagnosis, and these findings should not be interpreted as an indication for immediate therapeutic intervention. Similarly, rather than routinely lowering standardized diagnostic thresholds, a more appropriate approach may be to evaluate these low-level signals comprehensively in conjunction with clinical, hematological, and, when appropriate, molecular findings. Sub-threshold or very low-level JAK2 V617F results should not therefore be completely disregarded as nonspecific findings, particularly in patients with persistent blood count abnormalities or other clinical features suggestive of MPN, and careful hematological evaluation and appropriate longitudinal follow-up should be considered. Conversely, a negative or very low-level JAK2 V617F result should not be interpreted as definitively excluding an underlying hematological disorder.

This study also highlights the importance of using JAK2 V617F testing based on appropriate clinical indications in non-malignant conditions such as secondary polycythemia. Restricting test requests to clinically appropriate indications may contribute to more efficient diagnostic workflows and reduce unnecessary testing. Accordingly, targeted clinician education may be beneficial in supporting the use of JAK2 V617F analysis as a targeted diagnostic tool guided by clinical and hematological findings rather than as a routine screening test.

## Figures and Tables

**Table 1:** The Patients’ Baseline Clinical and Hematological Characteristics.

Baseline Characteristic	VAF Group 1 0.010–0.020%	VAF Group 2 0.021–0.040%	VAF Group 3 0.041–0.099%	*p* Value
Age (mean ± SD)	49.8 ± 16.9	52.1 ± 17.5	50.6 ± 16.8	0.621
Male sex	75.4% (*n* = 46)	78.6% (*n* = 55)	61.9% (*n* = 26)	0.174
Hematological malignancy	42.3% (*n* = 11)	26.9% (*n* = 7)	30.8% (*n* = 8)	0.285
Constitutional symptoms	50.8% (*n* = 31)	57.1% (*n* = 40)	71.4% (*n* = 30)	0.042 *
Splenomegaly	26.4% (*n* = 14)	16.4% (*n* = 10)	30.6% (*n* = 11)	0.807
Asthma/COPD	11.5% (*n* = 7)	7.1% (*n* = 5)	9.5% (*n* = 4)	0.670
Smoking	19.7% (*n* = 12)	34.3% (*n* = 24)	14.3% (*n* = 6)	0.748

* Overall comparisons among the three JAK2 V617F VAF groups were performed using Pearson’s chi-square test, as appropriate. For variables with a statistically significant *p*-value, post hoc pairwise comparisons were performed between Groups 1 and 2, Groups 1 and 3, and Groups 2 and 3. Bonferroni correction was applied to each set of three pairwise comparisons. The Bonferroni-adjusted *p*-values are reported in Section 3.

**Table 2:** Hematological Characteristics According to the JAK2 V617F Allele Burden Groups.

Hematological Parameters	The Entire Cohort Mean ± SD	VAF Group 1 0.010–0.020% Median (*n* = 61)	VAF Group 2 0.021–0.040% Median (*n* = 70)	VAF Group 3 0.041–0.099% Median (*n* = 42)	*p* Value
White Blood Cell Count	12.8 × 10^3^/µL	7.16 × 10^3^/µL	7.98 × 10^3^/µL	8.45 × 10^3^/µL	0.110
Hemoglobin (HG)	15.26 g/dL	16.6 g/dL	16.6 g/dL	15.65 g/dL	0.232
Platelet (PLT)	272 × 10^3^/µL	227 × 10^3^/µL	246 × 10^3^/µL	263 × 10^3^/µL	0.381
Lymphocytes (LYM)	4.08 × 10^3^/µL	2.13 × 10^3^/µL	2.12 × 10^3^/µL	2.43 × 10^3^/µL	0.686
Neutrophils (NEU)	8.68 × 10^3^/µL	4.07 × 10^3^/µL	4.55 × 10^3^/µL	4.95 × 10^3^/µL	0.445
Monocytes (MONO)	0.89 × 10^3^/µL	0.60 × 10^3^/µL	0.63 × 10^3^/µL	0.73 × 10^3^/µL	0.113
Eosinophils (EOZ)	0.29 × 10^3^/µL	0.13 × 10^3^/µL	0.10 × 10^3^/µL	0.14 × 10^3^/µL	0.446

Continuous variables were presented as median (IQR) or mean ± SD, as appropriate. Non-normally distributed hematological parameters were compared among VAF Groups 1, 2, and 3 using the Kruskal–Wallis test. Categorical variables were evaluated using Pearson’s chi-square. A *p* < 0.05 was considered statistically significant.

**Table 3:** A Comparison of Hematological Abnormalities across the JAK2 V617F Allele Burden Groups.

Variable	VAF Group 1 0.010–0.020%	VAF Group 2 0.021–0.040%	VAF Group 3 0.041–0.099%	*p* Value
Leukocytosis	23.0% (*n* = 14)	20.0% (*n* = 14)	47.6% (*n* = 20)	0.004
Erythrocytosis	65.6% (*n* = 40)	64.3% (*n* = 45)	52.4% (*n* = 22)	0.345
Thrombocytosis	9.8% (*n* = 6)	10.0% (*n* = 7)	21.4% (*n* = 9)	0.150
Neutrophilia	37.7% (*n* = 23)	48.6% (*n* = 34)	64.3% (*n* = 27)	0.030
Lymphocytosis	24.6% (*n* = 15)	22.9% (*n* = 16)	33.3% (*n* = 14)	0.450
Monocytosis	14.8% (*n* = 9)	28.6% (*n* = 20)	38.1% (*n* = 16)	0.024
Eosinophilia	27.9% (*n* = 17)	21.4% (*n* = 15)	31.0% (*n* = 13)	0.495

Overall comparisons among the three JAK2 V617F VAF groups were performed using Pearson’s chi-square test. Post hoc pairwise comparisons were subsequently performed between the two groups. Bonferroni correction was applied to the three pairwise comparisons for each variable, resulting in an adjusted significance threshold of *p* < 0.0167 (0.05/3). The *p*-values presented in the table correspond to the overall comparisons among the three groups.

## Data Availability

The data presented in this study are available on request from the corresponding author. The data are not publicly available due to privacy and ethical restrictions.
